# Is there a Chance to Promote Arteriogenesis by DPP4 Inhibitors Even in Type 2 Diabetes? A Critical Review

**DOI:** 10.3390/cells7100181

**Published:** 2018-10-22

**Authors:** Srinivasan Vedantham, Anna-Kristina Kluever, Elisabeth Deindl

**Affiliations:** 1 MedGenome Labs Ltd., Bangalore, Karnataka 560099, India; srinivasan.vedantham@gmail.com; 2 School of Chemical Biotechnology, SASTRA University, Thanjavur 613401, India; 3 Walter-Brendel-Centre of Experimental Medicine, University Hospital, LMU Munich, 80336 Munich, Germany; annakluever97@gmail.com

**Keywords:** cardiovascular disease (CVD), arteriogenesis, diabetes mellitus, dipeptidyl-peptidase-4 (DPP4) inhibitors, stromal-cell-derived factor-1 (SDF-1), gliptins

## Abstract

Cardiovascular diseases (CVD) are still the prevailing cause of death not only in industrialized countries, but even worldwide. Type 2 diabetes mellitus (type 2 DM) and hyperlipidemia, a metabolic disorder that is often associated with diabetes, are major risk factors for developing CVD. Recently, clinical trials proved the safety of gliptins in treating patients with type 2 DM. Gliptins are dipeptidyl-peptidase 4 (DPP4/CD26) inhibitors, which stabilize glucagon-like peptide-1 (GLP-1), thereby increasing the bioavailability of insulin. Moreover, blocking DPP4 results in increased levels of stromal cell derived factor 1 (SDF-1). SDF-1 has been shown in pre-clinical animal studies to improve heart function and survival after myocardial infarction, and to promote arteriogenesis, the growth of natural bypasses, compensating for the function of an occluded artery. Clinical trials, however, failed to demonstrate a superiority of gliptins compared to placebo treated type 2 DM patients in terms of cardiovascular (CV) outcomes. This review highlights the function of DPP4 inhibitors in type 2 DM, and in treating cardiovascular diseases, with special emphasis on arteriogenesis. It critically addresses the potency of currently available gliptins and gives rise to hope by pointing out the most relevant questions that need to be resolved.

## 1. Introduction

Cardiovascular diseases (CVD), such as coronary heart disease, cerebrovascular disease, and peripheral artery disease, are still the major cause of morbidity and death worldwide. For 2030, the World Health Organization (WHO) projected around 23 million deaths due to CVD [1]. Hyperlipidemia and diabetes mellitus are major risk factors for CVD by increasing atherosclerosis [2]. Moreover, collateral artery growth (arteriogenesis), which compensates for the functional loss of an artery due to atherosclerosis, is compromised in diabetes [3,4], a disease which is often associated with hyperlipidemia (see below). Accordingly, there is a pressing need for effective drugs to treat CVD, which also show positive effects in patients with diabetes, even with insulin resistance. In particular, these drugs should have the capacity to promote arteriogenesis, which is a tissue-, and even life-saving, process. Dipeptidyl-peptidase 4 (DPP4/CD26) inhibitors might present such drugs.

## 2. Arteriogenesis

The detrimental consequences of arterial stenosis, such as stroke, myocardial infarction, or peripheral artery disease, are a huge burden for affected patients worldwide. Occlusion of main blood conducting arteries results in severe ischemic damage of distal tissue, which might not be only associated with tissue damage, but even death. However, in the clinic, it is repeatedly observed that patients with occluded arteries show only minimal symptoms. These patients profited from arteriogenesis as a response to slow progressive stenosis [5]. Our whole body, not only the brain and the heart, but also the periphery, is pervaded with a collateral artery network. Upon slow progression of stenosis formation, blood flow is redirected into pre-existing collateral arteries bypassing the stenosing artery. Due to the increased blood flow, the endothelial cells of detouring arterioles experience elevated mechanical load, i.e. fluid shear stress [6], leading to their activation [7]. In contrast to angiogenesis, which is defined as the sprouting of capillaries, collateral artery growth not only involves the proliferation of endothelial cells, but also of smooth muscle cells. Recently, a mechanosensory complex consisting of vascular endothelial growth factor receptor 2 (VEGFR2), platelet endothelial cell adhesion molecule (PECAM)-1, and the adaptor protein vascular endothelial cell cadherin (VE-cadherin) has been identified [8]. It has been discussed for a long time whether the VEGFR2 ligand, vascular endothelial growth factor-A (VEGF-A), plays a role in arteriogenesis [9]. Whereas blocking of VEGFR2 significantly interfered with the process of arteriogenesis, administration of VEGF-A showed only little or no effect. We were recently able to show that the process of collateral artery growth is associated with elevated levels of VEGF-A [10], and that these levels of VEGF-A are not only necessary, but also sufficient to drive arteriogenesis. Accordingly, application of VEGF-A will not further promote the growth of collaterals, whereas blocking its cognate receptor, VEGFR2, dramatically interferes with vessel growth. As endothelial cells do not produce VEGF-A [11], the question arises, what is the source of VEGF-A. The endothelial glycocalyx consists of hyaluronic acid, glycoproteins, and proteoglycans, conferring it a negative charge. In the extended endothelial cell surface layer, plasma proteins and growth factors, among them VEGF-A, are stored [12]. Due to locally increased fluid shear stress, VEGF-A binds to VEGFR2, which involves Neuropilin-1 amplifying VEGFR2 signaling [13]. Thereupon, probably several cascades become activated in endothelial cells. Amongst them, the phosphatidylinositide 3-kinases (PI3K)/Akt pathway is relevant for increased expression levels of endothelial nitric oxide synthase (eNOS) and neuronal nitric oxide synthase (nNOS). Both isoforms are relevant for endothelial cell proliferation and are likely able to substitute for each other [10]. Moreover, VEGFR2/Neuropilin-1 signaling has been engaged in releasing the major ligand of the platelet receptor glycoprotein 1bα (GPIbα), the van Willebrand factor (vWF), from endothelial cells [14]. In recent investigations, we have demonstrated that GPIbα is essential for transient interaction of platelets with the endothelium [15], which is likely to trigger surface expression of P-selectin on platelets [16]. Upon binding of P-selectin to P-selectin glycoprotein ligand 1 (PSGL-1) on neutrophils, platelet-neutrophil aggregates (PNAs) are formed, resulting in neutrophil-NADPH oxidase 2 (Nox2) activation and translocation of urokinase plasminogen activator (uPA) on the surface of neutrophils [17]. Neutrophil uPA, together with endothelial cell uPA, whose expression is increased during the process of arteriogenesis [18], are relevant for paracellular transmigration and extravasation of neutrophils [19]. In the perivascular space, reactive oxygen species (ROS)- derived from neutrophil Nox-2 activate mast cells [17]. Mast cells, in turn, recruit further neutrophils by increasing the bioavailability of tumor necrosis factor α (TNF α). Neutrophils are a rich source of VEGF-A, the expression and release of which is mediated by midkine [10], enforcing VEGF-A signal transduction cascades resulting in endothelial cell proliferation and mast cell activation. In addition to neutrophils, activated mast cells also recruit T cells [17]. Moreover, by increasing the bioavailability of monocyte chemoattractant protein-1 (MCP-1), they recruit monocytes to the perivascular space [17]. Upon maturation to macrophages, these cells supply a variety of cytokines and growth factors—among them VEGF-A—to the growing vessel [20,21]. Altogether, arteriogenesis is a local inflammatory process, in which mast cells play an essential role by recruiting growth promoting leukocytes.

**Figure 1:**
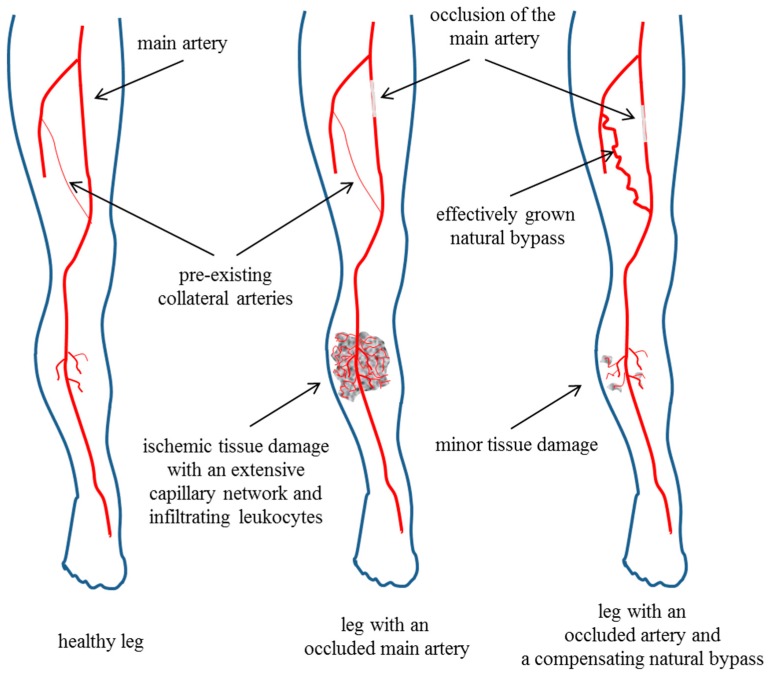
Arteriogenesis versus angiogenesis. The left picture displays a healthy human leg. In the left and the middle picture, a pre-existing collateral artery is shown. As arteries have the function to transport oxygenated blood to distal parts of the body—in the case of the femoral artery to the lower leg—occlusion of the femoral artery results in severe ischemic damage in the lower leg (middle picture). This is associated with extensive angiogenesis. However, the reason for capillary sprouting in this case is not to locally provide oxygen and metabolites—this is simply not possible as long as the feeding artery is occluded, or the bypassing pre-existing collateral has not yet been enlarged by growth—but to remove cell debris from tissue damaged by ischemia. The right picture shows a human leg with a completely occluded artery, which is bypassed by an effectively grown collateral artery compensating for the function of the occluded artery. Accordingly, there is minor ischemic damage in the lower leg with only little capillary sprouting. This picture reflects the tissue saving property of the process of arteriogenesis. Adapted from Chillo et al., 2016 [17] with the permission of Cell Reports.

Angiogenesis is also a local inflammatory process [22]. Whereas capillaries have the function to provide oxygen and metabolites for tissues locally, arteries transport oxygenated blood through the body. Accordingly, only arteriogenesis, the growth of pre-existing arteriolar connections to functional arteries, is capable to compensate for the loss an artery (Figure 1).

Patients with diabetes show a high prevalence of high-grade coronary atherosclerosis [23]. Unfortunately, in these patients, the growth of compensating coronary collateral arteries is severely impaired [24]. Peripheral artery disease is also a common complication in type 2 diabetes mellitus (type 2 DM) [25]. It is associated with lower extremity function, critical limb ischemia, and foot ulceration [26,27], and, due to impaired arteriogenesis [28], with limb amputation [29]. The long-term prognosis of patients with DM and peripheral artery disease is extremely poor, with a high mortality rate [30].

## 3. Glucose Uptake and the Metabolic Disorder Diabetes Mellitus

### 3.1. Incretins, Insulin, and the Adrenergic System

Glucose in chyme stimulates the release of the metabolic hormones, gastric inhibitory polypeptide (GIP) and glucose-dependent insulinotropic peptide-1 (GLP-1), from intestinal mucosa. Thereupon, these incretins augment the secretion of insulin, a polypeptide hormone, which is produced by beta cells of the pancreatic islets. This endocrine pancreatic activity is reciprocally regulated by the adrenergic system. While activation of the β_2_-adrenoreceptor (β_2_-AR) promotes insulin release [31], it is repressed when the α_2_-AR becomes activated [32]. In the blood, insulin reduces the blood glucose levels by stimulating cells to absorb glucose from the blood. Again, this process is potentiated through activation of β_2_-AR by adrenaline [33] and antagonized by noradrenergic α_1_-AR stimulation [34,35]. Moreover, insulin is a functional opponent of glucagon. Glucagon is a peptide hormone, which is released from the pancreas to the bloodstream upon stimulation of β-adrenergic receptors mainly due to hypoglycemia, protein rich food, and stress. In the liver, glucagon stimulates gluconeogenesis and glycogenolysis, resulting in increased blood glucose levels. Insulin and GLP-1 inhibit glucagon secretion, resulting in reduction of blood glucose levels. Binding of insulin to its receptor results in cross-talk with β_2_-AR, and β_2_-AR gene deletion results in hepatic insulin resistance [36]. Increased prevalence of insulin resistance in elderly patients with type 2 diabetes mellitus has been related to reduced expression of β_2_-AR [31,37,38]. Resistance to insulin is characterized by defects in muscle glucose uptake and hepatic glucose overproduction.

The adrenergic system has also been implicated in angiogenesis. While stimulation of α_1_-AR interfered with endothelial cell proliferation, migration, and tube formation, antagonizing the receptor showed the opposite effect. Moreover, blocking the receptor resulted in enhanced extracellular signal regulated kinase (ERK) activation and retinoblastoma phosphorylation, relevant for cell proliferation [39]. Deficiency of the β_2_-AR on endothelial cells, in contrast, impaired nuclear factor ‘kappa-light-chain-enhancer’ (NF-κB) activation and tube formation in vitro and angiogenesis in vivo [40]. However, further investigations are necessary to define the exact molecular mechanisms and signal transduction cascades associated with adrenoreceptor signaling in endothelial cells.

### 3.2. Diabetes Mellitus and Glucose-Dependent Insulinotropic *Peptide-1* (GLP-1)

Diabetes mellitus refers to a metabolic disorder resulting in high blood glucose levels due to either reduced insulin levels caused by destruction of insulin-producing beta-cells (type 1 DM), or insulin resistance (type 2 DM). Out of the two, type 2 DM is the prevailing form, with 95% of all DM cases being type 2 [41]. Moreover, about 85% of type 2 DM exhibit insulin resistance [42]. Besides hyperlipidemia, DM is a major risk factor for coronary and peripheral artery diseases [2].

Increasing insulin resistance elicits increased levels of free fatty acids in the blood. Accordingly, hyperglycemia in type 2 DM is often associated with hyperlipidemia, a metabolic disorder, which is characterized by high concentrations of triglycerides and low-density lipoprotein (LDL) cholesterol in the blood. Besides its action on insulin release, GLP-1 also regulates cholesterol and triglycerides. It reduces VLDL triglyceride production in the liver and regulates reverse cholesterol transport [43,44,45,46,47,48]. GLP-1 as well as GIP are rapidly inactivated by the enzyme, DPP4 [49], whose activity correlates with insulin resistance in type 2 DM [50,51].

Whether GLP-1 acts on hepatocytes, thereby directly exerting a function in the liver, is controversially discussed. Although there are data available showing the expression of the GLP-1 receptor (GLP-1R) on hepatocytes [52], these results were not confirmed by others [53]. As insulin resistance is a common problem in patients with type 2 DM, it is of major importance to clarify whether GLP-1 and, accordingly, GLP-1-based drugs (GLP-1 analogues, see below) have the capacity to exert their beneficial effects not only indirectly via insulin and glucagon, but also directly by binding the GLP-1R on hepatocytes. When treating healthy subjects with GLP-1 (45 pmol/kg per h), D’Alessio et al. found that GLP-1 improves glucose tolerance, and suggested that this is due to stimulation of insulin release and of insulin-independent glucose disposal [54]. In contrast, by infusing physiological postprandial levels of GLP-1 (0.4 pmol/kg per min), Seghieri et al. found a reduction in hepatic glucose production, but no effect on glucose disposal, and concluded that GLP-1 either directly inhibits hepatic gluconeogenesis or has a neutral effect [55]. Since GLP-1 is rapidly degraded by DPP4, it is not suitable as a drug to treat patients. Accordingly, DPP4–resistant mimetics of GLP-1 have been developed. These include exenatide [56], which is the synthetic form of the naturally occurring peptide, exendin-4 [57]. Interestingly, exendin-4 has been described to increase glucokinase enzyme activity in the liver independent of insulin (probably by acting on the hepatic GLP-1R) [58] and to improve insulin sensitivity [59]. For the latter, several mechanisms are discussed [60,61,62,63,64]. Since GLP-1 analogues are not applicable orally, non-peptide agonists would be desirable, which do not require self-injection by patients.

Recently, it has been shown that GLP-1 also stimulates nitric oxide (NO) production, thereby reducing blood pressure and the risk of atherosclerosis [65]. Indeed, it has been described that endothelial NO synthase (eNOS) is uncoupled in type 2 DM, thereby resulting in decreased levels of NO [66,67,68]. Interestingly, it has been demonstrated that blocking DPP4 activity gives rise to increased NO production, even independent of GLP-1, by promoting phosphorylation of endothelial NO synthase (eNOS) [69]. Orally applicable DPP4 inhibitors are currently used to treat type 2 DM (see below).

## 4. Molecular Functions of Dipeptidyl-Peptidase-4 (DPP4)

Proteases are involved in all kinds of physiological and pathophysiological processes, such as inflammation, fertilization, cell proliferation and death, tumor growth, and tissue remodeling [70]. DPP4 belongs to a class of specialized proteases, which cleave proline adjacent bonds. These proteases consist of (1) metallopeptidases, such as aminopeptidase P, carboxypeptidase P, and prolidase; and (2) three serine proteases, i.e. prolyloligopeptidase, DPP2, and DPP4 [70]. DPP4 belongs to prolyloliogopeptidases, which have evolved by independent convergent evolution [71,72]. A number of DPP4-like proteins are identified, which show either similarity to DPP4 activity, or are structurally related to DPP4, but enzymatically inactive. These proteins are called DPP4 activity and/or structure homologues (DASH) [73] and, for example, include fibroblast activation protein (FAP) [74], DPP4b [75], DPP6 [76], DPP7 [77], and DPP8 [78].

DPP4 is an intrinsic membrane glycoprotein that cleaves X-proline dipeptides from the N-terminus of polypeptides, however, it also exists in a soluble form [79]. Thus, DPP4 plays a major role in the regulation and activation of paracrine and autocrine as well as of extracellular endocrine peptides [80]. The protein is expressed in a variety of cells, although mainly in adipocytes [51], macrophages [81], T cells [82], endothelial cells [83], epithelial cells [84], and hepatocytes [85].

Beyond glucose control, DPP4 has a variety of functions, and not all of them are mediated by the enzymatic activity of the protein. The extracellular domain, which is involved in adenosine deaminase (ADA)/extracellular matrix binding, is exclusively responsible for the enzymatic activity of the enzyme [86]. Besides cleaving GLP-1 and GIP, both of which stimulate insulin secretion (see above), DPP4 also cleaves the regulatory peptides, glucagon-like peptide-2 (GLP-2), which plays a part in intestinal growth and function [87]; gastrin-releasing peptide (GRP) [88], which is involved in gastric acid secretion; and growth-hormone-releasing factor (GHRF), which stimulates growth hormone production [89]. However, DPP4 also modifies neuropeptides enzymatically. Among them are B-type natriuretic peptide (BNP), playing a role in vasodilation [90]; substance P, a neurotransmitter [91]; peptide YY, which is involved in electrolyte absorption in the colon [92]; and neuropeptide Y (NPY), which is linked to learning and memory [93]. Moreover, DPP4 cleaves a variety of chemokines, which are all involved in chemotaxis and leukocyte recruitment, including interferon-inducible T cell α chemoattractant (ITAC) [94], interferon-γ-induced protein-10 (IP-10) [94], Eotaxin [95], monokine induced by interferon γ (MIG) [96], macrophage derived chemokine (MDC) [97], regulated on activation normal T cell expressed and presumably secreted (RANTES) [98], granulocyte colony stimulating factor (G-CSF) [99], granulocyte monocyte colony stimulating factor (GM-CSF) [99], and stromal-cell-derived factor-1 (SDF-1) [94]. For an overview of the DPP4 mediated effects see Table 1.

Besides its enzymatic activity, DPP4 can interact with several other ligands. By binding to adenosine deaminase (ADA) [100], caveolin-1 [101], insulin growth factor receptor 2 (IGFR2) [102], and protein tyrosine phosphatase receptor type C (PTPRC, D45) [103], DPP4 positively regulates T cell co-activation. Moreover, it triggers T cell proliferation by binding to caveolin-1 and caspase recruitment domain-containing protein 11 (CARD11) [101]. It also promotes the proteolysis of the extracellular matrix and the migration of endothelial cells by interacting with separase [104]. Furthermore, DPP4 induces CD68 upregulation on monocytes by binding to caveolin-1 [105]. By associating with the Na^+^-H^+^ ion exchanger isoform sodium–hydrogen exchanger 3 (NHE3), DPP4 has been suggested to regulate NH3 activity and surface expression [106], and it is postulated that DPP4 also interacts with the thromboxane A2 receptor [107].

## 5. Cardiovascular Functions of Stromal-Cell-Derived Factor-1

The substrate of DPP4 SDF-1/CXCL12 plays a particularly important role in the cardiovascular system (see below). By interacting with its receptor, CXC-motive-chemokine receptor 4 (CXCR4), SDF-1 recruits leukocytes, such as neutrophils, monocytes, T-, and B cells, as well as other bone marrow derived CXCR4^+^ cells, such as stem cells and mast cells [17]. Askari et al. described SDF-1 as a key regulator involved in the homing of stem cells to ischemic myocardium, and showed that SDF-1 levels are already upregulated one hour after myocardial infarction, but return to baseline levels seven days later [108]. In rodent models, it was shown that overexpression of SDF-1 improved myocardial function after infarction and promoted revascularization [109,110]. Zhang et al. have shown that stem cells are not involved in the regeneration of cardiac myocytes after myocardial infarction, but that they play a beneficial paracrine role in cardiac myocyte survival and vascularization by supplying SDF-1 [111].

**Figure 2:**
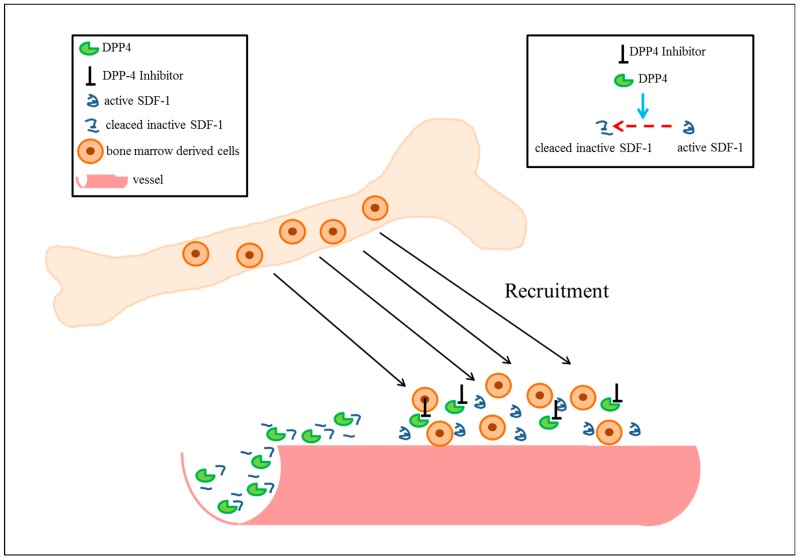
Recruitment of bone marrow derived cells by inhibition of dipeptidyl-peptidase 4 (DPP4). Stromal-cell-derived factor-1 (SDF-1) is cleaved by DPP4 (blue arrow) resulting in inactivation of the chemokine (red arrow). Blocking the enzymatic activity of DPP4 by specific (pharmacological) inhibitors preserves SDF-1. By binding to the receptor, CXC-motive-chemokine receptor 4 (CXCR-4), which is expressed on stem cells, leukocytes, and mast cells, SDF-1 locally recruits bone marrow derived cells, which in turn promote e.g. vessel growth (see Section 6.1).

As SDF-1 is cleaved and hence inactivated by DPP4 [112], it was suggested that inhibition of DPP4 activity and thus stabilization of SDF-1 might be a promising approach to treat cardiovascular and peripheral artery diseases (for an example see Figure 2).

## 6. DPP4 Inhibitors and Cardiovascular Diseases

### 6.1. DPP4 Inhibitors in Pre-Clinical Studies

Particularly due to the function of SDF-1 to recruit bone marrow derived cells, DPP4 inhibitors have been used to increase SDF-1 levels, aiming to improve CVD in pre-clinical animal studies. In several studies, the DPP4 inhibitor, Diprotin A, which is administered intraperitoneally (i.p.), has been applied. Referring to the studies of Christopherson et al. [112], who showed that Diprotin A increased the transmigration of progenitor cells towards an SDF-1 gradient, Zaruba et al. [113] performed studies in a murine model of myocardial infarction. This investigation showed that Diprotin A treatment decreased DPP4 activity in the myocardium (but not in the serum), which was associated with increased levels of SDF-1 and homing of CXCR-4^+^ stem cells, finally resulting in reduced cardiac remodeling, increased neovascularization, and improved myocardial function and survival. Similar results were obtained by Dingenouts et al. [114], who moreover showed that Diprotin A treatment results in a shift towards regenerative M2 macrophages in infarcted myocardium. We performed studies on a murine hindlimb model of arteriogenesis, a shear stress triggered process that relies on local recruitment of leukocytes, which promote the proliferation of endothelial- and smooth muscle cells of pre-existing collateral arterioles, finally resulting in natural bypass growth [5]. Our studies demonstrated that Diprotin A treatment increased SDF-1 levels in collaterals (but not in serum), which resulted in increased mast cell recruitment. In turn, mast cell recruited leukocytes (neutrophils, T cells, and macrophages) significantly enhanced arteriogenesis [17]. Using the same model of arteriogenesis, although with sitagliptin as a DPP4 inhibitor, Haverslag et al. also found an improved perfusion recovery in atherosclerosis prone ApoE-/- mice [115]. Interestingly, their results suggested that DPP4 inhibition showed no adverse side effects on atherogenesis and might even contribute to plaque stability. In accordance with our study, Haverslag et al. observed an increased accumulation of macrophages in the perivascular space of growing collaterals. Moreover, their results demonstrated an increased expression of the monocyte activation marker, CD11b, and of the SDF-1 receptor, CXCR-4, relevant for leukocyte activation and transmigration through activation of lymphocyte function-associated antigen-1 (LFA-1), and of very late antigen-4 and -5 (VLA-4, VLA-5) [116] on circulating monocytes. However, enhanced SDF-1 mediated CXCR-4 activation is also likely to induce NF-κB signaling in leukocytes [117] as well as phosphatidylinositol 3-kinase (PI3K), p44/42 mitogen-activated protein kinase (Erk 1 and Erk 2) [117], transforming growth factor-β (TGF-β), and tumor necrosis factor-α (TNF-α) [118] signaling in vascular cells, which are all relevant for effective collateral artery growth [10,119,120,121,122]. In a parallel study, it was shown by Krieger et al. that hydrogels locally releasing SDF-1 supported sustained natural bypass growth by recruiting M2 polarized macrophages [123], which have been shown to enhance arteriogenesis by promoting vascular remodeling [124]. This is an interesting finding, since Brenner et al. showed that sitagliptin treatment of mice (see below) mitigated atherosclerosis by priming monocytes into M2 macrophages [125]. Accordingly, these results strengthen the findings of Haverslag on atherogenesis in the mouse model of arteriogenesis in ApoE-/- mice.

In recent investigations, we were able to show that insulin treatment rescued arteriogenesis in streptozotocin-induced type 2 DM in mice by restoring leukocyte recruitment (unpublished own data). These data give rise to hope for treatment of type 2 DM patients with vascular occlusive diseases. Since the stabilization of GLP-1 through DPP4 inhibitors resulted in increased bioavailability of and sensitivity to insulin [126], and since DPP4 inhibitors have a positive effect on hypercholesterolemia, which also interferes with arteriogenesis [127], DPP4 inhibitors might not only restore arteriogenesis in type 2 DM patients, but even enhance it due to the local increase of SDF-1 levels. Moreover, DPP4 inhibitors evoked increased bioavailability of NO, relevant for proper arteriogenesis [10], which might also contribute to beneficial effects. Since G-CSF and GM-CSF are also substrates of DPP4, blocking its activity might additionally promote arteriogenesis since both factors were shown to enhance collateral artery growth when administered exogenously [128,129]. The importance of arteriogenesis has recently been demonstrated in a meta-analysis showing that high collateralization in patients with coronary artery disease (CAD) is associated with a 36% reduction of mortality risk [130]. Promoting natural bypass growth by drugs presents an elegant and non-invasive alternative to current clinical interventions, such as percutaneous transluminal angioplasty (PTA), percutaneous transluminal coronary angioplasty (PTCA), and bypass transplantation.

### 6.2. DPP4 Inhibitors in Clinical Studies

As DDP4 inhibitors represent a promising tool to treat patients with type 2 DM, several clinical studies were performed (for a short overview see Table 2). As diprotin A cannot be used for treating patients [131], other orally applicable DPP4 inhibitors, so-called gliptins, were applied. First results of meta-analyses of randomized controlled trials (RCTs), such as TECOS (Trial Evaluating Cardiovascular outcomes with Sitagliptin) [132], SAVOR-TIMI (Saxagliptin Assessment of Vascular Outcomes Recorded in Patients with Type 2 Diabetes Mellitus) [133], or EXAMINE (Examination of Cardiovascular Outcomes: Alogliptin vs. Standard Care in Patients With Type 2 Diabetes Mellitus and Acute Coronary Syndrome) [134], displayed a trend toward a lower incidence of major cardiovascular events (MACE), paving the way for further studies (recently reviewed in [135]).

All the subsequent trials showed that DPP4 inhibitors have a good safety profile. Due to the positive effects of DPP4 inhibitors on angiogenesis and arteriogenesis shown in pre-clinical studies, one would expect detrimental effects on retinopathy and nephropathy, which are caused by excessive vascularization [137] and are highly prevalent in patients with type 2 DM [137,138,139]. Yet the opposite was the case. Several clinical studies reported retino- as well as renoprotective effects of DPP4 inhibitors [140,141,142,143]. However, in terms of CVD, DPP4 inhibitors were not superior compared to placebo. This may be explained by several facts: (1) The trials were primarily designed to prove the safety of DPP4 inhibitors and not their superiority in terms of CVD outcomes; (2) the duration of these studies was probably too short (two to three years) to show any differences in CV (cardiovascular) outcomes; (3) that the positive effect in some patient subgroups may be compensated by zero (or even negative) outcomes in other subgroups. At this point, it is relevant to mention that the first trials only included patients with a rather low risk of CVD. Accordingly, the number of patients with surgical interventions, such as PTCA or intensive pharmacological treatment, was very low. This is an important point in terms of arteriogenesis. When the process of stenosis occurs (very slowly), the blood flow is redirected into pre-existing collateral arteries, triggering them to grow. However, if the stenotic artery is re-vascularized by PTA or PTCA, growing or grown collaterals are degraded as they are not needed anymore. Moreover, drugs, such as, for example, angiotensin-converting enzyme inhibitors, which degrade SDF-1 [144], might interfere with the positive effect of DPP4 inhibitors in arteriogenesis. Accordingly, new analyses are necessary, which take surgical and pharmacological treatment of patients into account; furthermore (4) the dose as well as the administration route might play a role. Recent studies on animal models using sitagliptin, which has been shown to be safe in the TECOS study, addressed this point (see Section 6.3).

### 6.3. DPP4 Inhibitors Revisited in Pre-Clinical Studies

Sitagliptin is administered orally in patients at a dose of 100 mg/d. The bioavailability is 87%, but 79% of the drug is excreted unchanged in the urine. Theiss et al. [145] performed a study on outcomes of myocardial infarction in mice using different dosages of orally administered sitagliptin ranging from 5 mg/kg/d to 500 mg/kg/d, and showed that only the treatment with 500 mg/kg significantly reduced DPP4 activity in the blood, whereby significantly increased numbers of bone marrow derived cells homing in the myocardium after infarction were already observed at a dose of 50 mg/kg. Treatment regimens with 500 mg/kg/d resulted in significantly reduced cardiac remodeling, increased capillary density, and improved myocardial function and survival of mice. The same results were obtained when the DPP4 inhibitor, vildagliptin, was used at an equal dosage. Brenner and co-workers, who also used 500 mg/kg/d of sitagliptin for oral treatment of mice, reported ameliorated atherosclerosis based on priming monocytes into M2 macrophages [125]. Moreover, they demonstrated that SDF-1, independent of GLP-1 action, shows a direct proliferative effect on endothelial cells [146], a process that is decisive for arteriogenesis [147].

Ghorpade et al. [148], recently published a study showing that oral administration of 35–40 mg/kg/d of sitagliptin significantly decreased plasma DPP4 activity in diet-induced obese (DIO) mice. The same result was obtained when mice were treated intravenously (i.v.) with a short hairpin RNA (shRNA) that specifically silenced DPP4 in the liver. However, in contrast to orally administered sitagliptin, only liver specific silencing of DPP4 decreased liver DPP4 protein levels and hepatocyte DPP4 activity.

Moreover, liver specific silencing of DPP4 lowered plasma insulin, improved glucose uptake, and suppressed insulin resistance in DIO mice. These data are in accordance with studies reporting that oral DPP4 inhibitors do not lower plasma insulin in insulin-resistant, hyperinsulinemic patients [149,150,151,152,153,154]. These facts throw a completely new light on orally administered DPP4 inhibitors in patients with type 2 DM in terms of rescuing or even enhancing the process of arteriogenesis. Several questions thus need to be addressed in pre-clinical studies.

### 6.4. Questions

As oral DPP4 inhibitors do not suppress insulin resistance, the first question is whether this is due to the oral application form or due to the dosage. GLP-1 receptor agonists, such as exenatide or semaglutide, used to increase insulin sensitivity in patients, are administered subcutaneously [155,156]. DPP4 increases insulin resistance by enzymatically cleaving GLP-1. However, it still remains to be elucidated whether insulin sensitivity is additionally promoted by a non-enzymatic function of DPP4. Therefore, the question arises of whether gliptins really are the best choice to treat insulin resistance. Another question is whether gliptins work equally well in rodents and humans concerning the specific recognition of DPP4 or whether differences in efficacy are due to differences in species. The next question is whether currently available DPP4 inhibitors reach all desired target organs (including the liver). Accordingly, the final question arises: Do we have to develop a new class of DPP4 inhibitors? Last, but not least, the data on patients in terms of improvements of CVD need to be (re-)addressed, considering whether the overall surgical and pharmacological treatment of each subject might interfere with the process of arteriogenesis.

## 7. Conclusions

DPP4 inhibitors have been shown to be safe in clinical trials and might efficiently promote arteriogenesis even in patients with type 2 DM. However, several problems need to be resolved before we will be able to efficiently promote arteriogenesis even in patients with type 2 DM.

## Figures and Tables

**Table 1:** DPP4 activity.

Function	Protein	Result of DPP4 Cleavage
Regulatory peptides	GLP-1	Inactivation
	GLP-2	Inactivation
	GIP	Inactivation
	GRP	Inactivation
	GHRF	Inactivation
Neuropeptides	BNP	Activity reduced
	Substance P	Inactivation
	Peptide YY	Receptor specificity
	NPY	Receptor specificity
Chemokines	ITAC	Inactivation
	IP-10	Inactivation
	Eotaxin	Inactivation
	MIG	Altered cell type specificity
	MDC	Inactivation
	RANTES	Inactivation
	G-CSF	Inactivation
	GM-CSF	Inactivation

Abbreviations: Glucagon-like peptide 1 (GLP-1); glucose-dependent insulinotropic peptide (GIP); glucagon-like peptide-2 (GLP-2); gastrin-releasing peptide (GRP); growth-hormone-releasing factor (GHRF); B-type natriuretic peptide (BNP); neuropeptide Y (NPY); interferon-inducible T cell α chemoattractant (ITAC); interferon-γ-induced protein-10 (IP-10); monokine induced by interferon γ (MIG); macrophage derived chemokine (MDC); regulated on activation normal T cell expressed and presumably secreted (RANTES); granulocyte colony stimulating factor (G-CSF); granulocyte monocyte colony stimulating factor (GM-CSF); stromal-cell-derived factor-1 (SDF-1).

**Table 2:** Meta-analyses of clinical trials using DPP4 inhibitors.

Study	Endpoint
SAVOR-TIMI, Saxagliptin Assessment of Vascular Outcomes Recorded in Patients with Type 2 Diabetes Mellitus [133]	cardiovascular death, nonfatal myocardial infarction, nonfatal stroke
TECOS, Trial Evaluating Cardiovascular Outcomes with Sitagliptin [132]	cardiovascular death, nonfatal myocardial infarction, nonfatal stroke
EXAMINE, Examination of Cardiovascular Outcomes: Alogliptin vs. Standard Care in Patients with Type 2 Diabetes Mellitus and Acute Coronary Syndrome [134]	cardiovascular death, nonfatal myocardial infarction, nonfatal stroke
CAROLINA, Cardiovascular Outcome Study of Linagliptin v.s. Glimepride in Patients with Type 2 Diabetes Mellitus [136]	cardiovascular death, nonfatal myocardial infarction, nonfatal stroke, unstable angina pectoris

Reviewed 2018 by S. J. Scheen, see [135].
